# Adenoma of Soft Palate

**Published:** 1877-05

**Authors:** 


### ARTICLE VIII.

### *Adenoma of Soft Palate.*

Dr. A. H. Smith, at a recent meeting of the N. Y. Pathological Society, presented a tumor removed by operation from the soft palate of a patient who had come to him from the interior of the State with a diagnosis of malignant disease. The growth projected but slightly into the mouth, but the whole of the space behind the velum on the right side, and the adjacent pharnyx was occupied. The growth was about the size of a butternut. About two years ago, the patient first discovered a small growth in the soft palate, which gradually increased until it became the size of an almond. At that time the physician who saw the case advised the application of caustic. The result was the formation of an eschar which, when separated, gave issue to a considerable amount of glary mucus-like fluid. The wound healed, and for a time it seemed as if the difficulty had entirely disappeared. A year ago last December the growth again commenced to show itself, and continued to increase until Dr. Smith saw him. He formed the opinion that the tumor was of that variety first described by Nelaton as adenoid. Extirpation was advised and performed accordingly. The operation was very simple, and consisted merely in the division of the mucous membrane over the tumor, and shelling it out with the finger pressed from behind. The only embarrassment was in consequence of the cicatrix, which had to be divided with the scissors. The patient during the operation was placed in Ross's position, with the head over the edge of the table. The recovery was rapid and complete.

Dr. Smith remarked that the clinical features of these tumors were slowness of growth, painless character, freedom from attachment to mucous membrane, and their non-liability to pass beyond the raphe of the soft palate.—*Medical Record.*

